# tESA: a distributional measure for calculating semantic relatedness

**DOI:** 10.1186/s13326-016-0109-6

**Published:** 2016-12-28

**Authors:** Maciej Rybinski, José Francisco Aldana-Montes

**Affiliations:** Departamento LCC, University of Malaga, Campus Teatinos, Malaga, 29010 Spain

**Keywords:** Bioinformatics, Semantic relatedness, Semantic similarity, Distributional linguistics, Knowledge extraction, Explicit semantic analysis, Biomedical semantics

## Abstract

**Background:**

Semantic relatedness is a measure that quantifies the strength of a semantic link between two concepts. Often, it can be efficiently approximated with methods that operate on words, which represent these concepts. Approximating semantic relatedness between texts and concepts represented by these texts is an important part of many text and knowledge processing tasks of crucial importance in the ever growing domain of biomedical informatics. The problem of most state-of-the-art methods for calculating semantic relatedness is their dependence on highly specialized, structured knowledge resources, which makes these methods poorly adaptable for many usage scenarios. On the other hand, the domain knowledge in the Life Sciences has become more and more accessible, but mostly in its unstructured form - as texts in large document collections, which makes its use more challenging for automated processing. In this paper we present tESA, an extension to a well known Explicit Semantic Relatedness (ESA) method.

**Results:**

In our extension we use two separate sets of vectors, corresponding to different sections of the articles from the underlying corpus of documents, as opposed to the original method, which only uses a single vector space. We present an evaluation of Life Sciences domain-focused applicability of both tESA and domain-adapted Explicit Semantic Analysis. The methods are tested against a set of standard benchmarks established for the evaluation of biomedical semantic relatedness quality. Our experiments show that the propsed method achieves results comparable with or superior to the current state-of-the-art methods. Additionally, a comparative discussion of the results obtained with tESA and ESA is presented, together with a study of the adaptability of the methods to different corpora and their performance with different input parameters.

**Conclusions:**

Our findings suggest that combined use of the semantics from different sections (i.e. extending the original ESA methodology with the use of title vectors) of the documents of scientific corpora may be used to enhance the performance of a distributional semantic relatedness measures, which can be observed in the largest reference datasets. We also present the impact of the proposed extension on the size of distributional representations.

## Background

### Introduction

A rapid growth in scientific publishing has been observed in recent years. Thanks to online resources, the access to this literature seems easier and quicker than ever, but often the sheer volume of potentially relevant articles makes it extremely difficult for the end user. However, working with these large text collections may actually result in the development of methods for automatic semantic processing and annotation that could greatly improve intelligent data access. This paper focuses on the problem of calculating distributional semantic relatedness based on a large document corpus by leveraging the semantics from different sections of the corpus elements (i.e. by making an explicit use of the semantics of titles of scientific papers). Semantic relatedness is a metric that can be assigned to a pair of labels in order to represent the strength of the relationship of the concepts described by those labels. The automated calculation of the metric is the building block for numerous semantically enhanced data processing techniques such as: word sense disambiguation [1] (used for matching word contexts to the best word senses), text summarization [2] (used for evaluating cohesion of the lexical chains) and information retrieval [3] (incorporated in the query-document ranking method). Similar applications of relatedness and similarity (which is a narrower concept) metrics within the scope of Life Sciences include entity–entity relationship extraction [4, 5], semantic search [6] and redundancy detection in clinical records [7]. An overview of applying semantic similarity to the problem of comparing gene products is discussed in [8]. In [9] the authors discuss the application of a relatedness measure as an approximation of semantic similarity in the biomedical domain.

The methods for calculating semantic relatedness can be roughly divided into two main groups: those that rely entirely on a specialized and structured knowledge-rich resource (e.g. [10–12]), and distributional measures that rely on implicit statistical features of a large document collection (e.g. [13, 14]). With the increased popularity of using Wikipedia as a Knowledge Base (KB) for semantic relatedness estimation this division has become much less clear, as Wikipedia combines the features of both worlds. It does implicate a structure, as it comprises a set of topic-oriented and categorized entries, which are also interconnected with hyperlinks. It can also be treated as a large collection of documents, as it contains over 2M articles with at least 150 words each.

In this paper we focus on corpus-based distributional methods for calculating semantic relatedness and we present a new measure, which can be applied in the biomedical domain without having to rely on specialized knowledge rich resources. In our approach, which is an extension of a well-established state-of-the-art method, we superimpose the semantics of different sections of documents (i.e. we make ‘additional’ use of the titles of scientific articles). We demonstrate that our method slightly outperforms other state-of-the-art approaches while relying on the very limited structure of the documents within the corpus (only abstracts and titles from the Medline corpus [15] are used in the best performance setting).

### Related work

There is a significant body of work devoted to biomedical ontology-independent (to a certain degree) relatedness measures that rely on context vectors, i.e. the immediate neighborhoods of the phrases/words throughout a document corpus, e.g. [16]. In the method presented in [16], context vectors are created using a *sliding window* technique, that is, scanning through contexts of a certain size throughout the entire corpus of documents in order to find words that co-occur with certain terms or phrases of interest. In order for this technique to be employed, the authors use a predefined set of these terms/phrases, i.e. *SNOMED CT*. SNOMED CT is the largest medical vocabulary collection, with over 400K systematically organized concepts with their lexical representations and additional information. In the method presented in [16], the distributional representations are created for each SNOMED CT concept by adding word vectors of tokens relevant to respective concepts. Despite the fact that the approach uses additional resources (SNOMED, Mayo Clinic Thesaurus), the relatedness calculation depends on the corpus co-occurence distribution, without referring explicitly to the ontological structure of SNOMED. Both in [17], and more recently in [9], a similar approach has been used with a different set of resources. The main feature that sets the method presented in our paper apart is that it does not need pre-existing concept descriptions (such as those of SNOMED CT) in order to produce the relatedness score.

As mentioned briefly, there is a large group of methods that use Wikipedia as a knowledge resource/document collection, some examples include [18–20]. Most of these measures exploit Wikipedia-specific features such as links or categories. Nonetheless, Wikipedia as a resource (at least currently) is too general in nature for many Life Sciences applications. Therefore, from our perspective, the methods that treat the data more like a generic document collection seem more appealing, the most notable example being Explicit Semantic Analysis (ESA) [21]. In ESA, the input texts are represented by a vector, in which each element corresponds to a Wikipedia article. Values of each of the elements are determined by the importance of the input text to the contents of each article, i.e. i-th element of the vector for a word or a phrase will be determined by the importance of the word within the i-th Wikipedia article (formal description of the method is provided further on in this paper). The relatedness between the inputs is calculated as the cosine similarity between those vectors.

Numerous extensions of ESA have been proposed, many of which combine the original approach with the Wikipedia-specific features, through concept-to-concept feature/similarity matrices, e.g. [22–24]. Some of those extensions, e.g. NESA [25] (Non - Orthogonal ESA), also provide variants that are generic enough to be used with any document collection. The aim of NESA is to leverage inter-document similarity in the calculations. In our measure the input is modeled in a way similar to ESA, but we propose an extension so as to capture the feature based similarity between sets of documents. However our method is much more resource efficient than NESA, which facilitates handling a large corpus of documents.

In the biomedical domain there have also been several attempts to use Wikipedia based methods, recent examples include [26] and [27]. The former presents an application of the ESA methodology to a KB extracted automatically from MedLine Plus corpus in the context of semantic relatedness. The latter uses ESA inspired methodology with yet another KB in the context of document classification.

As we have previously argued [28], results comparable to those of state-of-the-art methods can be obtained by approximating the context vectors with the vectors extracted from the relatively small sample of best-fit documents from a moderately sized PMC open subset corpus [29]. We now expand on these conclusions in combination with an ESA inspired approach to achieve better results, coverage and independence from the specific parameters of the algorithm, which was one of the drawbacks in our previous approach. The new method takes advantage of a larger document collection (Medline), but performs well with only the abstracts and titles available.

Within the NLP community, so called *word embedding* methods have received much attention. In these techniques words or phrases from the original corpus are mapped to low dimensional vectors through language modelling and/or feature learning. One of the most widely discussed representative of this group, *word2vec* [30] is a group of methods that use neural networks for unsupervised training of a model that either predicts a context given a word, or predicts the word given a context. Application of word2vec in biomedical settings is presented in a recent study [31].

There is also a significant body of work related to KB-based semantic relatedness measures which use highly specialized resources, described in a detailed overview in [32] and [33]. KB-based methods are useful wherever an adequate domain knowledge model can be used to compute semantic relatedness. In [34] the authors showcase the performance of a wide spectrum of ontology based Information Content (IC) methods, which use SNOMED CT as a knowledge resource. The IC measures use an ontological structure (positions of concepts in the ontology, distance between them, number of sub-concepts, etc.) to compute a semantic score between a pair of concepts. Our method, although dependent on a specific corpus, does not rely on high level KB representations of the domain, which makes it more flexible and easier to adapt to non-standard use cases.

### Contributions

Here we present *Title vector Explicit Semantic Analysis* (tESA), a novel approach for approximating word-based semantic relatedness, which uses a document corpus as its only source of background knowledge. The tESA method itself is an extension of ESA, based on using two sets of vectors corresponding to different sections of the documents of the corpus. Together with the experiments detailing its performance, tESA is our primary contribution.

Additionally, we present a parallel evaluation of the original ESA methodology in the same settings (corpora and reference standards). To the best of our knowledge it is the first time that the ESA implementation has been evaluated in such detail within the biomedical domain.

In the Methods section we present a detailed description of ESA, tESA and the experimental evaluation. We also highlight the distinguishing design features of tESA by comparing it to other corpus-based methods. Then, in the Results and discussion section we present the results obtained through the evaluation, compare them to other state-of-the-art methods and discuss some of the implications. In the final Conclusions section, apart from presenting the final remarks, we also outline possible lines of future work.

## Methods

In this section, we firstly explain the basic concepts that will help clarify the design of the tESA method. We then provide a short description of the original ESA method and then we introduce the tESA method, while outlining the main differences between the two.

### Basic notions

The black-box view of a semantic relatedness approximation system is fairly simple - the system takes two input texts (also referred to as inputs) and returns a relatedness approximation (score). The inputs can be texts of variable length, typically single words or short phrases are considered.

The actual processing involves the inputs and a collection of documents - referred to as the corpus. We use a term ‘document’ to denote a semistructured textual resource that forms part of this collection, i.e. a document can be formed by a number of sections; here, we focus on a simplified case of documents consisting either of titles and abstracts or titles and the fulltext body (depending on their availability in various document collections included in the evaluation).

As mentioned, our method is based on a distributional vector representation of input texts. As is common in many distributional linguistics algorithms, we use certain variations of the *tf-idf* (term frequency, inverse document frequency) weighting scheme as the underlying vector model for text representation. So, at the most basic level, prior to relatedness calculations, any texts (inputs, document abstracts, titles) are modeled as *tf-idf* weighted vectors. Term frequency is the number of times a given term appears within the scope of a certain text (i.e. certain section of a document), while inverse document frequency is defined in the context of a specific document collection:

1 $$ \text{idf} (t, D, f) = \log{\frac{N}{|{d_{f} \in D : t\in d_{f}}|}},  $$

where *D* denotes a certain corpus of documents, *N* denotes size of the corpus, *t* denotes the term and *d* a document, *f* denotes a section of documents from the corpus and *d*_*f*_ a text of the section *f* of a document *d*. Those elements lead us to the formula for *tf-idf*:

2 $$  \text{tfidf}(t, d_{f}, D) = \text{tf}(t,d_{f}) \times \text{idf}(t,D,f)  $$

The equation presents a basic implementation of *tf-idf* weighting, whereas within our approach we use slightly different variants. For modelling abstracts the in-built Lucene [35] scoring function is used. It uses a document length normalization factor, a square root norm for the *tf* factor and a square norm for the *idf* factor. For titles we assume *tf* equals 1 whenever a term appears within the title and zero otherwise. Nonetheless, the basic idea is that within a vector for a single document higher weights are assigned to terms that either appear more often within the document or are less common throughout the entire corpus. When creating the vector representation of text using the *tf-idf* scheme, vectors are assembled by placing a weight corresponding to each of the document’s terms at the position corresponding to the term, so the dimensionality of the model is given by the number of unique words present in the section of the documents throughout the collection. Therefore the vector space is of a very high dimension, while the actual vectors are normally sparse.

It is worth noting, that, given a corpus and a specific section of its documents, the vector representation can be created for any text, regardless of whether the text belongs to the corpus or not. This representation will obviously differ depending on the choice of the corpus and the section. This notion is typically used in vector-based information retrieval (IR), where most relevant documents are found for an input query and a field or a combination of fields of an index, where fields correspond to sections and index to the corpus. Commonly, to decide whether a document fits the query, one can compare the vector representing the query with the vector representing the section of a document. We use *cosine similarity* as the basic tool for pairwise vector comparison. This applies to word-based *tf-idf* vectors and extends to other types of vectors, as explained further on in this section. For a pair of *n* element vectors $\overline {A}$ and $\overline {B}$ the cosine similarity is defined as follows:

3 $$  cosine(\overline{A},\overline{B}) = \frac{\sum \limits_{i=1}^{n} A_{i} B_{i}}{\sqrt{\sum \limits_{i=1}^{n} {A_{i}^{2}}}\sqrt{\sum \limits_{i=1}^{n} {B_{i}^{2}}}}  $$

### Text preprocessing

We use standard Lucene mechanisms for pre-processing of texts prior to the tf-idf vectors computations. Texts are transformed to lowercase and stopwords (words that occur very commonly, but provide little or no semantic information, e.g. the, of, at, a, etc.) are eliminated. Numbers are also eliminated and non-alphanumeric characters (e.g. ‘-’) are normalized. In case of the titles, we also disregard words that appear in less than 3 different documents of the respective corpora.

### ESA

These basic notions lead us to the more complex one of a *doc vector* (also referred to as *concept vector* in the original ESA paper [21]), which is the central building block of ESA. In the ESA method the doc vectors are used to provide a distributional representation of the inputs. The relatedness is then approximated for a pair of inputs by comparing their doc vectors. Cosine similarity is used to obtain the numeric result of this comparison. By a doc vector of an input *q* we mean a vector in which the value of an i-th element is calculated as a cosine similarity between: (a) the tf-idf vector representing the input *q* w.r.t. the IDF values calculated for the abstracts of the corpus; (b) tf-idf weighted vector representing an abstract of an i-th document of the corpus ^1^. It is worth noting that the dimensionality of the doc vector is given by the size of the corpus. Ttf-idf vector $\overline {q}_{abstract}$ represents an input *q* w.r.t. the statistics (i.e. IDF) derived from the abstract section of the corpus’ documents. We can define the doc vector $\overline {q}_{D}$ as a vector of weights *w*_*i,q*_, where

4 $$ w_{i,q} = cosine\left(\overline{abstract}_{i}, \overline{q}_{abstract}\right)  $$

where $\overline {abstract}_{i}$ denotes the tf-idf vector of the abstract for the *i-th* document from the N document corpus. In the original method a corpus of Wikipedia articles is used, along with their text contents. In this paper, apart from the original Wikipedia-based implementation, we also present experiments with domain-focused corpora.

In practical implementations it is enough to consider a set of *M* highest scores within the vector, as the tail of N-M values are either zeroes or have little impact on further processing. As such, ESA methodology can also be explained in information retrieval terms, with the input treated as a query and the results represented with a doc vector of non-zero values at M most significant elements. Those values, in a most basic tf-idf weighted vector space model representation, are given with the formula for *w*_*i,q*_. This intuitive explanation of ESA might clarify the step-by-step processing of tESA, presented further on in this section.

### tESA

It can be observed, that a corpus with documents that have more than one section can be used to establish more than one independent vector space, i.e. a corpus with documents that consist of titles and abstracts can be used to create a vector space of titles and a vector space of abstracts. Creation of a doc vector involves the vector space of abstracts to determine the weights/elements at positions corresponding to certain documents. Nonetheless, the doc vector itself is expressed in a yet another space of dimensions (of documents, rather than words). The main idea behind tESA is to create a similar vector expressed in a different vector space, i.e. one with notably fewer dimensions - a vector space of document titles. The *tESA vector* is a doc vector transformed through a multiplication by the column matrix of tf-idf vectors of titles (which means term-document matrix of title-associated tf-idf weights). The matrix represents the vector space model of the document titles. By tf-idf vectors of titles we refer to word-based tf-idf representations of individual titles of documents, while a tESA vector is a distributional representation of an input text, much like a doc vector in ESA. *C* denotes the column matrix of tf-idf vectors of titles; *C*_*ji*_, which denotes the element of j-th row and i-th column of *C* (which therefore corresponds to the title of the i-th document and j-th term of the title vector space), is given by (see Eq. 2):

5 $$ C_{ji} = {tfidf}(k_{j}, d_{title}(i), D),  $$

where *d*_*title*_(*i*) denotes the text of the title of the i-th document and D denotes the corpus of documents and *k*_*j*_ denotes the j-th term of the title vector space.

Given the matrix C defined above, let $\overline {q}_{T}$ denote a tESA vector of input *q*, while $\overline {q}_{D}$ denotes the doc vector of input q. The tESA vector $\overline {q}_{T}$ is defined as follows:

6 $$ \overline{q}_{T} = C \overline{q}_{D}  $$

This means, that using the Eq. (4) the j-th element of $\overline {q}_{T}$, *q*_*T*__*j*_, corresponding to a j-th row of the matrix C (an thus to the j-th term of the title vector space), is given by:

7 $$ {t_{T}}_{j} = \sum \limits_{i=1}^{N} cosine\left(\overline{abstract}_{i}, \overline{q}_{abstract}\right) \times C_{ji},  $$

where $\overline {abstract}_{i}$ denotes a tf-idf vector of the abstract of the i-th document and $\overline {q}_{abstract}$ denotes a tf-idf representation of the input q in the vector space of document abstracts. A j-th element of the tESA vector is therefore defined as a weighted sum of tf-idf weights of the j-th term (of the titles vector space) over the corpus of the document titles. This sum is weighted with the input-abstract cosine similarities from the doc vector.

As mentioned, in our implementation for the title vector space, we assume that *tf*(*k*_*j*_,*title*_*j*_)=1 if term *k*_*j*_ is present in the j-th title, otherwise the value of the tf term is 0. Additionally, to reduce the computations, in our implementation we calculate the tESA vector from a doc vector truncated at M of its most significant elements, as: (a) the tail values have little impact on the final results; (b) most commonly the doc vector will have fewer than M non-zero values anyway (which is discussed in the next section of this paper).

**Fig. 1:**
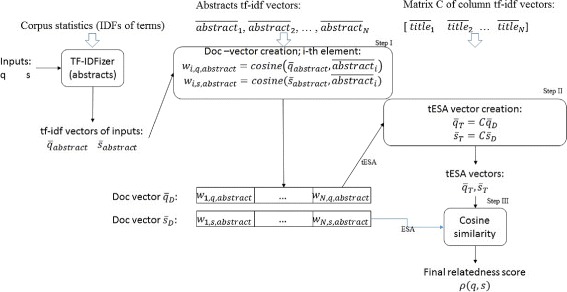
Overview. Overview of the method’s components

As displayed in Fig. 1, the processing of our method can be divided into three main steps:

- **I** Finding doc vectors of both inputs, truncated at M highest-value elements
- **II** Calculation of the tESA vectors for each of the inputs (see Eq. 5).
- **III** Using the tESA vectors to compute the relatedness approximation as the cosine similarity between the tESA vectors.

Under information retrieval terminology, we use the input text as a query for the abstract/fulltext based vector space model. Results of this query (scores for each of the individual documents, M values at the most) are represented by the doc vectors. In ESA we would use the doc vectors as the final representations of the inputs, meanwhile in tESA we perform an additional calculation. In other words, we transform the doc vectors to tESA vectors using the title vector space of the corpus and the formula of Eq. 6. Therefore, the resulting vector will have non-zero weights at positions corresponding to the vocabulary of titles of the documents in which the input terms appear within the abstracts. Additionally, we promote meaningful terms from the titles (through IDF), especially in the context of documents, in abstracts of which the input terms play a prominent role (modeled with the doc vector elements, here used as a prior). We expect this additional computational effort to provide an improvement on two levels: (a) an improvement in the quality of the results and (b) using ‘smaller’ representation vectors to model inputs. When it comes to improving the quality of the results, our expectations are based on the fact, that statistically it is likely that sets of titles of similar/related documents will share some part of the vocabulary. Our approach adds another level of intrinsic similarity between document sets, i.e. the input terms are related not only if they appear in the same abstracts, but also if the sets of abstracts they appear in share common features (title vocabulary). Our expectation of ‘smaller’ representations can be derived directly from two assumptions. Firstly, the dimensionality of the vector space of titles is much smaller when compared to the dimensionality of the vectors used in ESA (e.g. in the case of Medline the difference is of 300K compared to 14M). Secondly, using very short tf-idf word vectors to represent titles (the vectors are truncated to represent only the ‘top-idf’ vocabulary), combined with the expectation that some title vocabulary will overlap between documents, should result in representation vectors with fewer non-zero elements than the doc vectors. Both hypotheses, (a) and (b) are evaluated in the experiments.

### Design differences: tESA vs other distributional approaches

On a conceptual level the processing in our method is similar to ESA, except that in ESA the relatedness approximation is calculated directly as the cosine similarity of the doc vectors. The direct application of the ESA approach will also be discussed. As mentioned, the tESA vectors were designed to take advantage of inter-document similarity, by expressing the doc vector in the title vector space, in which the documents, or more importantly groups of documents, may share common features. XESA and NESA also benefit from the use of inter-document similarity but in an explicit manner, through the use of the document-to-document similarity matrix. The NESA approach uses an *N*×*N* sized dense document similarity matrix, which requires costly preprocessing and significant resources for runtime processing. The authors of XESA also contemplate the use of a truncated similarity matrix.

ESA and tESA provide a flexibility and efficiency advantage over approaches such as those presented in [16] and [17] and their extensions. Specifically, they use corpus statistics instead of relying on contex window word counts, which means that the new distributonal representations can be created without having to actually ‘scan’ through all the documents that contain the input terms, so the cost of creating the representation vectors is much lower.

Word embeddings (i.e. word2vec) have the advantage of using dense representation vectors of relatively low dimension (typically around 200), which makes those methods computationally appealing. However, the use of machine learning to pre-train the model hinders the flexibility of those methods to a certain degree. For example, switching from unigram to bigram inputs would require either re-training of the entire model or using some kind of composition strategy involving unigram vectors (addition, multiplication), while ESA and similar methods can be adapted relatively easily or need no adapting at all, depending on the actual implementation.

tESA can also be presented as an extension of the method presented in [28]. The previous approach uses a much smaller M to limit the number of relevant documents even further. Furthermore, it does not distinguish the importances of those documents, i.e. the representation vector was created simply by adding the M most important tf-idf truncated vectors of fulltext documents (not their titles). The extensions that differentiate tESA from the original method at the design level can therefore be summarized as follows: increased size of M, use of a vector transformation (see Eq. (5)) and use of title vectors instead of fulltext/abstract vectors. These changes might seem minor, but they actually represent an important change of focus, from an attempt to capture the sample of most relevant vocabulary to represent an input, to modeling the distribution an input ‘generates’ over a title vocabulary of a corpus.

### Experiments

The tESA method was designed to work with the Medline baseline corpus, which provides us with over 14M abstracts with corresponding titles. In addition, the methods were tested with different document collections, which included PMC Open Access (PMC OA) and Wikipedia articles. A summary of the corpora used in the experiments is presented in Table 1.

**Table 1:** Presentation of the general characteristics of the corpora used in the experiments

	MEDLINE	PMC OA	Wikipedia
Size	14073912	1024890	3807314
Type	Scientific	Scientific	Encyclopedic
Documents	Abstacts and titles	Mostly fulltext + abstracts + titles	Fulltext + titles
Snapshot date	Autumn 2015	September 2015	December 2015
Token count [M]	2531,14; 264,84	3684,89; 15,8	2434,55; 11,13
Unique token count [M]	3,85; 1,24	35,57; 0,48	12,53; 0,98

Token counts and unique token counts are expressed in millions. These statistics are collected for raw texts (before preprocessing) and raw corpora (e.g. there might be an uneven number of titles and abstracts in Medline). For each corpus and count type we provide two metrics - of the documents’ textual contents (abstract or full articles) and titles. The statistics are included to highlight the compositional differences between the corpora

The reference datasets used in the experiments were: mayo101 [36], mayo29c, mayo29ph [16], umnsrsRelate, umnsrsSim [37]. Each of the datasets represents a separate experiment, in which a group of annotators rated pairs of concepts for semantic relatedness (mayo101, mayo29c, mayo29ph, umnsrsRelate) or similarity (umnsrsSim). The datasets contain a list of pairs with a single consensus score. The consensus score available in the reference datasets was achieved by calculating an average score over multiple annotators. It is important to note that mayo29c and mayo29ph are high-agreement sets, rated by medical coders and physicians respectively. The mayo101 dataset consists of 101 concept pairs rated by a group of professional medical coders from Mayo Clinic. The remaining two datasets, i.e. umnsrsRelate and umnsrsSim, contain clinical concept pairs rated for similarity/relatedness by a group of medical residents. The latter two also include a standard deviation calculated for each pair of the labels, which can be used to approximate an inter-annotator agreement on each of the average scores. We use this feature to demonstrate the performance of the methods under discussion on high-agreement subsets of these two datasets. The size and other features of the reference datasets are summarized in Table 2.

**Table 2:** Presentation of the general characteristics of the datasets used in the experiments; number of pairs and distinct items describe the size of the datasets; the focus of the dataset column contains the information on the type of relationship captured in the reference results

Dataset	No of pairs	Distinct items	Reference	Focus of the dataset	Annotators	Scale	ICC(2,1)
umnsrsSim	566	375	[37]	Similarity	Residents	0 - 1600	0.47
umnsrsRelate	587	397	[37]	Relatedness	Residents	0 - 1600	0.5
mayo101	101	191	[36]	Relatedness	Medical coders	1 - 10	0.5
mayo29c	29	56	[16]	Relatedness	Medical coders	1 - 10	0.78
mayo29ph	29	56	[16]	Relatedness	Physicians	1 - 10	0.68

The ICC (2,1) presents interclass corelation coefficient, which provides an objective measure of inter-annotator agreement; the issues of inter-annotator reliability are covered in more detail in the corresponding reference papers

In the experimental evaluation of an automated measure, the pairs of labels from the reference dataset are treated as inputs. In most cases each input is a single word, although there are two-word inputs as well. For a list of pairs of inputs a list of relatedness scores is generated by the system. This list is then compared to the list of average scores generated by human annotators. The performance of the methods in approximating human judgement was measured as the Spearman’s rank correlation coefficient, as the problem can be seen as one of ordering the concept pairs within each dataset by their relatedness, i.e. both the consensus score and the approximation system rank the pairs within each reference dataset from the most related to the least related (by assigning scores). The performance has been measured for our implementation of ESA and tESA and is evaluated against other state-of-the-art methods, which, to the best of our knowledge, represent the best results reported in the literature.

Additionally, due to the nature of the methods, each pairing of a dataset and corpus may be associated with a certain *recall* value, which provides information on how appropriate the corpus is for the benchmark. Recall in our setting is defined as a ratio of the number of inputs with a representation to the total number of distinct items from a given dataset. It therefore gives the percentage of inputs that are present in each of the corpora, which means that they can be assigned a distributional representation.

Our experiments involved three methods: ESA, tESA, and the method presented in [28]. Each of the methods was evaluated with a combination of three different corpora. Additionally, we also compared them to the best results reported in the literature. NESA and XESA were not present in the evaluation, largely due to the high computational cost involved in creating an *N*×*N* similarity matrix for a corpus as large as Medline. Furthermore, our early experiments with a truncated similarity matrix actually caused an important performance drop compared to the original ESA setup with the same domain-focused corpus, which might indicate a high corpus sensitivity of the method and is is briefly discussed in the following section.

As stated, the quality of the methods is measured as a rank correlation with the reference scores produced by human annotators. In order to compare the performance of two methods we effectively compare the correlations they produce w.r.t. a specific reference sample of limited size. To provide a full perspective on our results, we evaluate the statistical significance of correlation comparisons using a methodology presented in [38]. Specifically we construct a 0,95 confidence level *confidence intervals* (CI) for dependent overlapping correlations (as for a pair of methods, both of them produce their correlation against the same reference dataset). This test allows us to refute, under the assumed confidence level, the null hypothesis of the two correlations being equal. As our main goal is to evaluate tESA, we test the statistical significance of tESA correlations vs those of other methods. We used [39] as a practical guide to implement the statistical test.

## Results and discussion

**Fig. 2:**
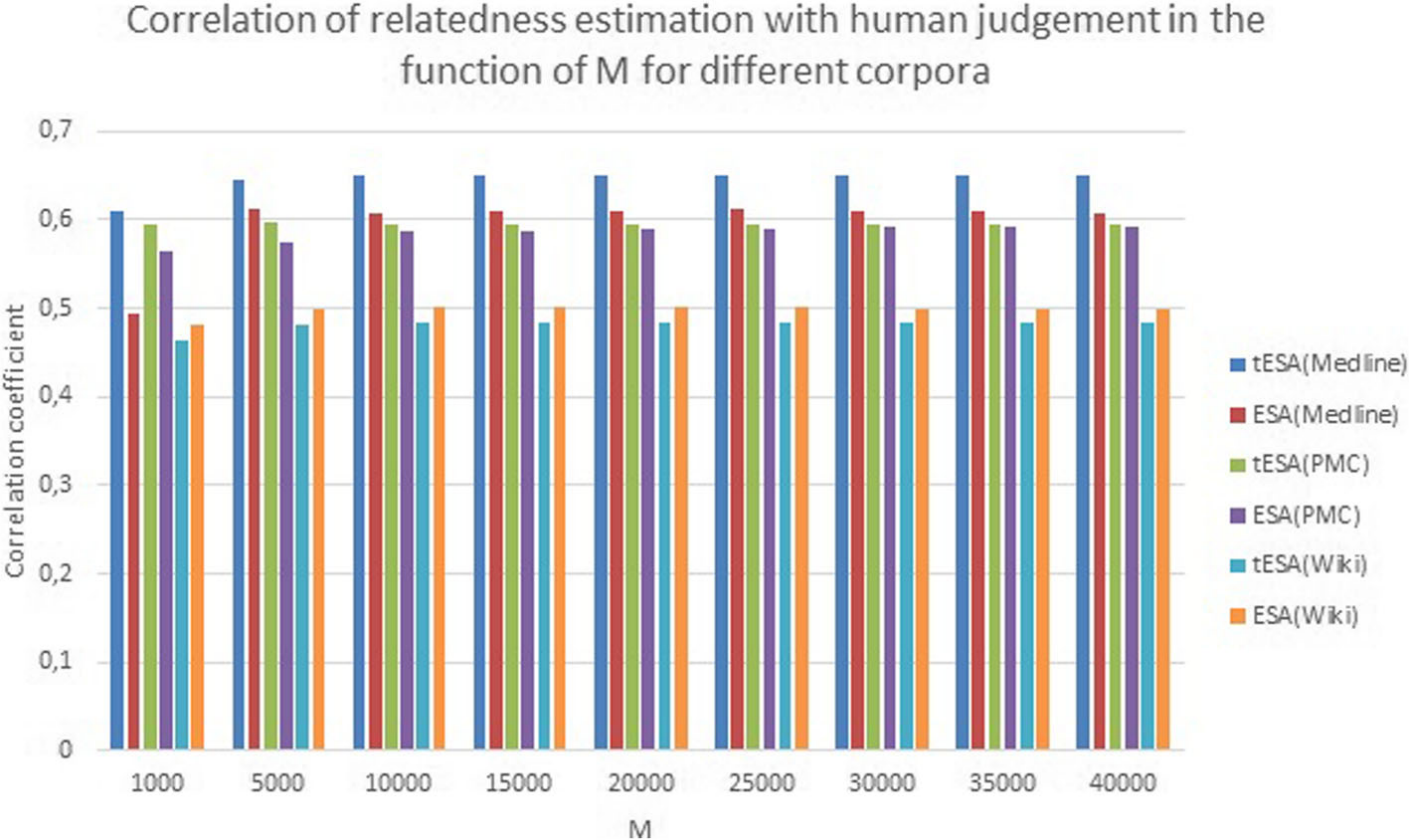
Performance changes for different M (cutoff limit for a maximum number of documents considered in the distributional representation). The figure shows the correlation with human judgement of ESA and tESA with different corpora in the function of M; the values were obtained for umnsrsRelate dataset

Table 3 shows the scores obtained with ESA, tESA, and the method presented in [28], with different corpora, for each of the reference datasets. The table also features the best reported score for each of the datasets. The results for tESA and ESA were obtained for *M=10000*, so each doc vector has non-zero values at, at most, 10000 positions (corresponding to the highest scoring documents). This value of the M parameter has been selected as a possibly small value for optimal performance of all setups/methods included in the evaluation - Fig. 2 shows how the results depend on the values of *M* for ESA and tESA with different corpora on the umnsrsRelate dataset.

**Table 3:** Overview of the results for different experimental settings - corpus and benchmark pairs; ESA and tESA runs with M=10000 and DS (the method described in [28]) runs with M=200 and cutoff at 0,02 (robust parameters, that can be expected to provide decent results in different experimental settings)

Corpus	Method	umnsrsRelate	umnsrsSim	mayo101	mayo29ph	mayo29c
	ESA	0.608	0.621	0.546	0.835	0.734
Medline	tESA	**0.649**	**0.639**	0.549	0.783	0.687
	DS	0.46	0.438	0.511	0.483	0.493
	ESA	0.588	0.597	0.543	0.855	0.75
PMC	tESA	0.595	0.607	0.484	0.796	0.7
	DS	0.574	0.626	0.504	0.738	0.673
	ESA	0.501	0.5	0.548	0.822	0.722
Wiki	tESA	0.484	0.484	0.502	0.801	0.755
	DS	0.444	0.463	0.413	0.627	0.597
Best reported (citation)		0.54 [28]	0.58 [28]	**0.6** [28]	0.84 [16]	**0.9** [34]

The table row for best reference results has been compiled with results reported in the domain literature for the respective datasets, regardless of the type of method used to achieve those results. Best reported results for umnsrsRelate, umnsrsSim and mayo101 were attained with specific parameter combinations in our experiments (presented in [28]), whereas for the two smaller datasets the best results were previously obtained with knowledge-rich methods (distributional and IC-based respectively for mayo29ph and mayo 29c). Updated best results are highlighted with bold font

**Fig. 3:**
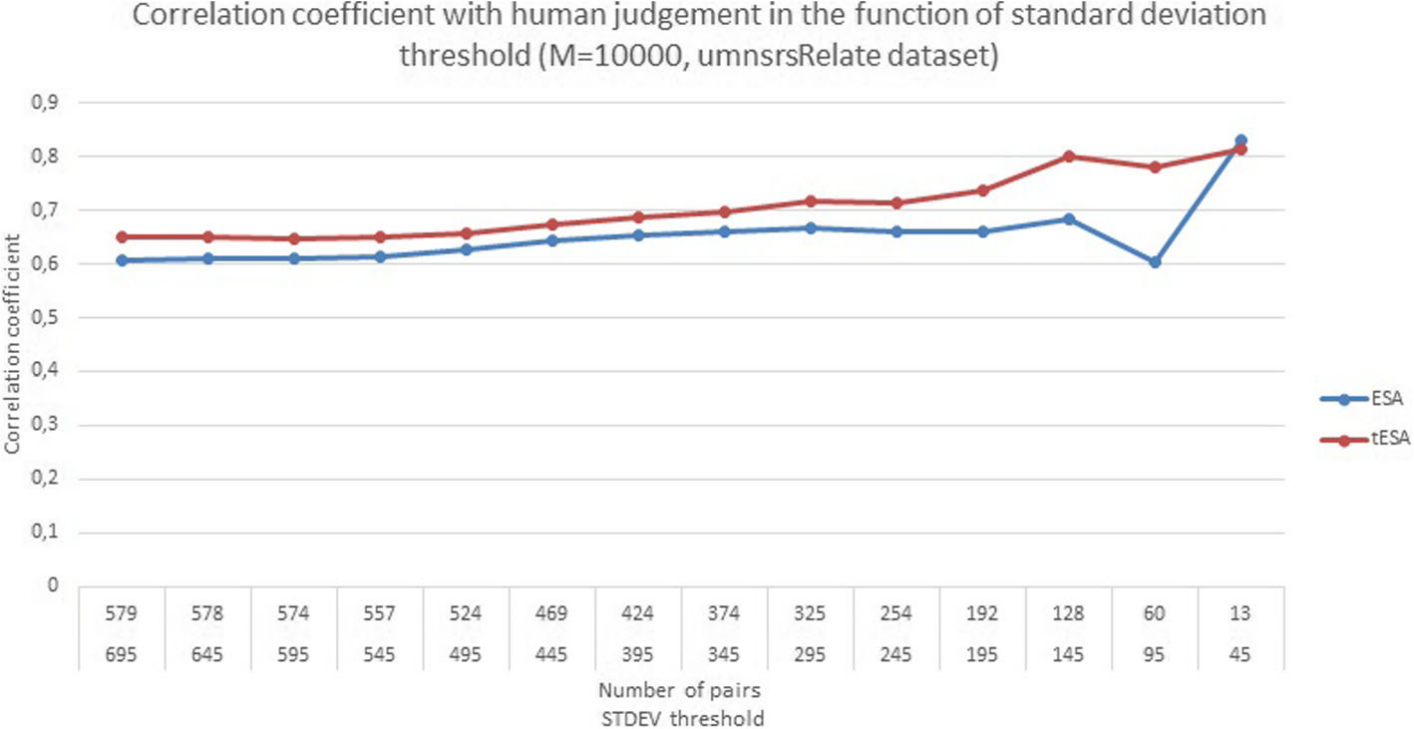
Performance in the function of increased inter-annotator agreement - umnsrsRelate. The figure shows the correlation with human judgement of ESA and tESA in the function of decreasing threshold for standard deviation, which is used to model the inter-annotator agreement, calculated for the umnsrsRelate reference dataset

**Fig. 4:**
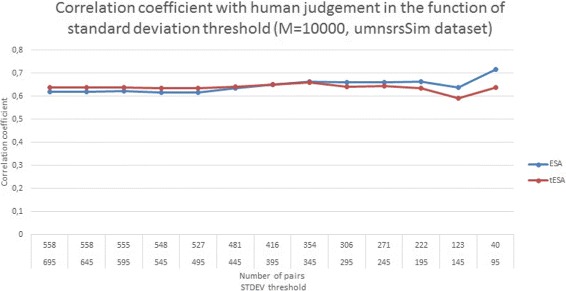
Performance in the function of increased inter-annotator agreement - umnsrsSim. The figure shows the correlation with human judgement of ESA and tESA in the function of decreasing threshold for standard deviation, which is used to model the inter-annotator agreement, calculated for the umnsrsSim reference dataset

Figure 3 presents the correlation coefficient obtained by the methods set up with the Medline corpus in the function of inter-annotator agreement for the umnsrsRelate dataset. For each run the dataset had a standard deviation threshold decreased in order to exclude the low agreement portions of the datasets. The data presented in Fig. 3 indicates that both ESA and tESA provide more accurate results for the sets that were more agreed upon by the human annotators. Although this seems intuitive, the improvement of the ranking in the function of inter-annotator agreement indicates that the method does provide a decent approximation of human judgment particularly w.r.t. the difficulties in reaching a correct score for the same pairs of inputs which seemed problematic for human annotators. In the case of a similar experiment performed on the umnsrsSim dataset, see Fig. 4, the link between the IAA and the quality of the results does not seem to be evident for tESA (which begins to show a decrease in performance at some point), while for ESA the performance decreases initially and begins to improve at a certain point. Considering that there is little evidence (only two experiments) it is difficult to reach a definite conclusion. There is a possibility. that the results presented in Fig. 4 are due to the fact that the umnsrsSim dataset is focused on semantic similarity, which is a narrower concept than semantic relatedness.

As shown in Table 4, all corpora provide similar recall values, with the highest values for Medline and lowest for Wikipedia. In other words, the datasets contain information on a similar percentage of inputs, so the differences in performance of the methods set up with different datasets will be related to the quality/precision of the information coverage rather than to its range.

**Table 4:** Recall for different dataset-corpus pairs. Recall is measured as a ratio of unique items (single input labels) represented by non-zero vectors to the total number of unique items in their respective datasets. As mayo29ph and mayo29c contain the same set of item pairs, the recall is identical for both datasets

Dataset	Medline	PMC	Wiki
umnsrsRelate	0.985	0.977	0.95
umnsrsSim	0.989	0.981	0.963
mayo101	0.957	0.951	0.929
mayo29	0.982	0.982	0.982

Table 5 shows the results of the statistical significance testing for pairs of experimental runs. We show which correlation differences from Table 3 are statistically significant w.r.t. a 0.95 confidence interval. The table lists CIs which indicate statistical significance of the comparisons, i.e. only CIs that do not include zero are presented.

**Table 5:** Statistical tests (confidence intervals) for differences between correlations reported in Table 3

tESA config	Other method	Dataset	CI	Comparison
Medline	DS (Medline)	mayo29ph	(0.09; 0.59)	+
Medline	ESA (Medline)	umnsrsRel	(0.003; 0.09)	+
Medline	ESA (PMC)	umnsrsRel	(0.025; 0.099)	+
Medline	ESA (Wiki)	umnsrsRel	(0.097; 0.2)	+
Medline	DS (Medline)	umnsrsRel	(0.13; 0.25)	+
Medline	DS (PMC)	umnsrsRel	(0.026; 0.12)	+
Medline	DS (Wiki)	umnsrsRel	(0.15; 0.26)	+
Medline	tESA (PMC)	umnsrsRel	(0.02; 0.09)	+
Medline	tESA (Wiki)	umnsrsRel	(0.11; 0.22)	+
Medline	ESA (PMC)	umnsrsSim	(0.004; 0.08)	+
Medline	ESA (Wiki)	umnsrsSim	(0.09; 0.19)	+
Medline	DS (Medline)	umnsrsSim	(0.14; 0.26)	+
Medline	DS (Wiki)	umnsrsSim	(0.11; 0.24)	+
Medline	tESA (Wiki)	umnsrsSim	(0.1; 0.21)	+
PMC	DS (Medline)	mayo29ph	(0.1; 0.61)	+
PMC	ESA (Wiki)	umnsrsRel	(0.04; 0.15)	+
PMC	DS (Medline)	umnsrsRel	(0.07; 0.2)	+
PMC	DS (Wiki)	umnsrsRel	(0.096; 0.21)	+
PMC	tESA (Wiki)	umnsrsRel	(0.06; 0.16)	+
PMC	ESA (Wiki)	umnsrsSim	(0.056; 0.16)	+
PMC	DS (Medline)	umnsrsSim	(0.1; 0.24)	+
PMC	DS (Wiki)	umnsrsSim	(0.09; 0.2)	+
PMC	tESA (Wiki)	umnsrsSim	(0.07; 0.18)	+
Wiki	DS (Medline)	mayo29c	(0.04; 0.55)	+
Wiki	DS (Medline)	mayo29ph	(0.11; 0.62)	+
Wiki	DS (Wiki)	mayo29ph	(0.01; 0.41)	+
Wiki	ESA (Medline)	umnsrsRel	(-0.18; -0.07)	-
Wiki	ESA (PMC)	umnsrsRel	(-0.15; -0.05)	-
Wiki	DS (PMC)	umnsrsRel	(-0.16; -0.025)	-
Wiki	ESA (Medline)	umnsrsSim	(-0.19; -0.086)	-
Wiki	ESA (PMC)	umnsrsSim	(-0.16; -0.06)	-
Wiki	DS (PMC)	umnsrsSim	(-0.21; -0.07)	-

The CIs were constructed for pairs of correlations involving at least one tESA setup. The table provides all the information necessary to track the CI back to Table 3, i.e. the corpus of the tESA method, the method (and corpus) to which the tESA results are being compared and the reference dataset. We also provide the CI itself, additionally indicating if the result is positive or negative

A quick glance at Table 3 reveals that both methods, i.e. tESA and ESA, surpass the existing methods on the two larger datasets, with the improvement being more evident in the case of tESA and the umnsrsRelate dataset (which is also evident in Table 5). This gain is less evident for the smaller datasets, nonetheless the ESA method paired with the PMC OA corpus provides a result which is better than the previously known best score. Additionally, the mayo29 datasets contain a very small data sample and mayo101 is only of moderate size, so it seems reasonably safe to assume that they are somewhat less reliable or at least more prone to incidental variations (which also shows in Table 5). Nonetheless, the scores achieved on mayo29 benchmarks seem to be comparable with several well established KB-based relatedness measures (refer to the evaluation presented in [34]).

Also, tESA and ESA are only outscored by the previous method for a specific combination of runtime parameters for a specific dataset. They do however seem to display more robustness, both in terms of parameter and corpus variations, i.e. they outperform the original method method presented in [28] on sub-optimal (consensus) settings used in Table 3. Furthermore, data presented in Fig. 2 suggest that both ESA and tESA perform consistently through a range for *M* values, so little corpus specific optimization for *M* is necessary (for the samples between 10K-40K, at 5K interval, range for neither of the methods exceeded 0,005). Obviously the value of M is still corpus dependent to some extent, i.e. it is best to avoid cutting off the ‘significant’ portions of the vectors. The data presented in Fig. 2 suggests that setting the value of M well above the average vector length works well, while keeping the size of long-tailed vectors (which represent very common tokens) under the limit. The M value of 10K was chosen for the main experiments, as it does not seem to hinder the performance of any of the method-corpus combinations.

Table 6 shows the mean number of non-zero vector elements throughout the reference datasets for ESA and tESA set-up with each of the corpora. Although tESA does require more processing to obtain a vector representation of an input (the method does the same as ESA, and then more, i.e. the computation of tESA vectors using the C matrix), the data shows that one can reasonably expect tESA vectors to have fewer non-zero values, which is especially evident in the case of the optimal Medline-based configuration. Additionally, tESA vectors are also less dimensional, as the titles contain fewer unique tokens (see Table 1) than the total number of documents in each of the corpora considered in our evaluation. These features account for an advantage of tESA over ESA, especially in scenarios where the costly part of the method can be delegated to a one time pre-processing effort. In other words, once the distributional representations have been computed, tESA is faster than ESA with two out of three corpora. Most importantly, it is more efficient in handling the representations extracted from Medline, which is the largest of the corpora and also provides the best-performance setting.

**Table 6:** Average vector ‘length’

	Medline	PMC	Wiki
tESA	3222,7	3547,4	535,8
ESA	4579,4	3391,9	751

The table shows an average of non-zero elements in tESA and ESA vectors, calculated throughout reference datasets for each of the corpora

From the perspective of the corpus choice, it can be argued that ESA-related methods rely on domain-adequacy of the entire corpus (thus the slight drop in performance for Wikipedia), but could also benefit from a larger document collection (increase in performance for Medline over PMC), all of which is consistent with the conclusions drawn in [40]. On the other hand, the method presented in [28] apparently depends more on the quality of individual documents, i.e. PMC’s full research papers return better results than Wikipedia articles and Wikipedia articles still give better results than abstracts in the Medline collection. This can be explained by the fact that the ESA-related methods, with high enough values of M, rely on the distribution of words throughout the collection. Whereas, the method presented in [28] relies on the presence of a small sample of documents from which a decent representation of the input can be retrieved. Bearing this in mind, one should note that the quality of each method is closely related to a combination of its intended use and available document collection.

The ESA methodology paired with the Wikipedia corpus is essentially an implementation of the ‘original’ ESA with a cutoff, so it provides an important baseline for other methods to be compared against. This baseline score is surpassed by ESA combined with domain specific corpora (Medline/PMC) on all benchmarks with the exception of mayo101, where the difference is statistically insignificant. tESA provides significantly better results than the ‘original’ ESA baseline for the two larger datasets. It also provides a better result for the mayo101 dataset, but the gain is statistically insignificant.

When comparing the performances of ESA and tESA, tESA seems to provide better results (at least for the most relevant benchmarks) when the methods use domain-oriented collections. One possible explanation is that the titles of scientific articles are simply more descriptive than those of Wikipedia. At the same time, the Wikipedia titles are usually short and contain discriminative tokens (almost like identifiers), and those tokens are sometimes accompanied by a broad categorical description (e.g. Medicine) intended for human disambiguation, which in the presented settings may increase noise. We believe that fine tuning the extraction method for title representation could improve tESA even to the point of achieving results more comparable with ESA with both methods using Wikipedia as the document corpus. Nonetheless using a document collection with more descriptive titles seems to be a safer choice when it comes to improving performance.

The results obtained both with tESA and ESA (especially with the Medline corpus) seem ecouraging given the results presented recently in [31]. Both tESA and ESA seem to achieve better results when evaluated against the two largest benchmarks than all the methods discussed in the study, while performing at least comparably to the best ones on the smaller reference datasets, although a deeper statistical analysis would be needed to provide more perspective. It is worth noting however, that both tESA and ESA operate on much larger structures (vectors) than some of the methods presented in the cited evaluation (e.g. word2vec-trained word embedding), which means that ESA-based approaches might be less appropriate for large scale tasks.

The approach used in tESA is similar to that used in the NESA methodology in the sense that it is aimed at leveraging the inter-document similarity. In NESA this is achieved by the explicit usage of a similarity matrix for all the documents, while in tESA it is done through the creation of the representation vectors as described in the Methods section. In other words, NESA and XESA contemplate leveraging the actual document-document similarity, while in tESA we assume that sets of documents might share common vocabulary features. The advantage of tESA is that it can be directly applied to larger corpora, as it needs a representation vector per word or document (depending on the actual implementation) and the target vector space is relatively small, while NESA requires storing a dense similarity matrix of an *N* ×*N* size. In [22], the use of a truncated matrix is contemplated, however our initial experiments with the truncated cosine similarity matrix have shown decreased performance and increased processing and preprocessing times when compared to tESA and ESA, which might point to an issue with the adaptability of the approach to domain-specific corpora and the specificity of the concepts within the evaluation datasets (especially when we compare it with the length and coverage of biomedical journal papers). As the task of adapting the similarity based ESA extensions is an independent research problem (which might be or not be feasible), it has been left to be considered in our future work, as outlined below.

Obviously, the tESA model is limited in terms of representing the inter-document similarity (as it does not reflect the similarity of actual document-document pairs), it does however seem to benefit from the intrinsic characteristics of the titles of the scientific papers. Nonetheless, our impression is that relatedness methods could be further enhanced by experimenting with the mapping and the target representation space. The goal of further work should therefore be to provide a better similarity modelling within the target representation space. We believe that this could be achieved by: (A) an intelligent approach towards extracting more informative representations from full texts/abstracts, (B) using NESA-like distribution based representations obtained for titles. With respect to (A) it has to be noted that preliminary experiments with the parameters of the approach presented in [28] (increasing the query size, decreasing the cutoff threshold) did not provide satisfactory results, probably due to the amount of noise introduced in the representations, therefore research thread (A) will center on finding a representation extraction method that maximizes information content, while reducing noise. The line of research related to (B) will focus on providing representations that do not lead to dimensionality problems and can be adapted to the biomedical domain, and comparing their performance with the NESA-like approaches.

## Conclusions

In this paper we have presented a new, robust method for computing lexical semantic relatedness for biomedical use - tESA. The approach uses a vector space of titles of scientific articles combined with ESA principles. We have also provided a side-by-side comparison of tESA and ESA, the latter method having not been evaluated as thoroughly in similar experimental settings. Both methods were reviewed with direct benchmarks, i.e. their ability to approximate human judgement was assessed. The algorithms outperfomed other state-of-the-art methods in the largest-to-date datasets used to evaluate biomedical semantic relatedness and similarity, with the original tESA method gaining a slight advantage.

Also, we have demonstrated that tESA uses ‘smaller’ and more dense vectors than ESA, so it might be a better fit in cases where vector computation cost (which is higher in tESA) is less important than the cost of online computations.

The results obtained with both tESA and ESA seem to be on par with the other state-of-the-art methods, a recent study [31] being a good point of reference.

The results obtained in our evaluation seem to indicate that the performance of the method can be optimized by choosing a correct background corpus, i.e. a domain oriented corpus of documents will provide a quality improvement in assessing domain-oriented relatedness. The baseline score of the ‘original ESA’ has been surpassed by both methods on the two largest (and thus more statistically significant) reference datasets.

We believe that the approach and detailed evaluation that we have presented may be a good fit wherever semantic relatedness approximation is a necessity, especially within subdomains that lack a detailed KB domain model, but are well covered in the scientific literature. Guidelines to tuning and applicability of the discussed methods have also been presented here. Finally, two interesting lines for future research have been outlined, both of which we hope to pursue in the near future.

## Endnote

^1^ The method actually uses either abstracts or full articles, depending on the features of the actual corpus, as explained further on.

## Abbreviations

ESA: Explicit semantic analysis
KB: Knowledge base
NESA: Non-orthogonal explicit semantic analysis
PMC: PubMed Central, also refers to PubMed Central Open Access document corpus
tESA: Title vector explicit semantic analysis
Tf - idf: Term frequency inverse document frequency
